# Trajectories of reported sleep duration associate with early childhood cognitive development

**DOI:** 10.1093/sleep/zsac264

**Published:** 2022-11-10

**Authors:** Shirong Cai, Elaine Kwang Hsia Tham, Hai-Yan Xu, Xiuju Fu, Rick Siow Mong Goh, Peter D Gluckman, Yap-Seng Chong, Fabian Yap, Lynette Pei-chi Shek, Oon Hoe Teoh, Joshua J Gooley, Daniel Yam-Thiam Goh, Michael J Meaney, Nora Schneider, Anne Rifkin-Graboi, Birit F P Broekman

**Affiliations:** Singapore Institute for Clinical Sciences, Agency for Science, Technology and Research (A*STAR), Singapore, Singapore; Human Potential Translational Research Programme, Yong Loo Lin School of Medicine, National University of Singapore, Singapore, Singapore; Singapore Institute for Clinical Sciences, Agency for Science, Technology and Research (A*STAR), Singapore, Singapore; Institute of High Performance Computing, Agency for Science, Technology and Research (A*STAR), Singapore, Singapore; Institute of High Performance Computing, Agency for Science, Technology and Research (A*STAR), Singapore, Singapore; Institute of High Performance Computing, Agency for Science, Technology and Research (A*STAR), Singapore, Singapore; Singapore Institute for Clinical Sciences, Agency for Science, Technology and Research (A*STAR), Singapore, Singapore; Liggins Institute, University of Auckland, Auckland, New Zealand; Singapore Institute for Clinical Sciences, Agency for Science, Technology and Research (A*STAR), Singapore, Singapore; Department of Obstetrics and Gynaecology, Yong Loo Lin School of Medicine, National University of Singapore, Singapore, Singapore; Department of Paediatric Endocrinology, KK Women’s and Children’s Hospital, Singapore, Singapore; Singapore Institute for Clinical Sciences, Agency for Science, Technology and Research (A*STAR), Singapore, Singapore; Department of Paediatrics, Yong Loo Lin School of Medicine, National University of Singapore, National University Health System, Singapore, Singapore; Khoo Teck Puat- National University Children’s Medical Institute, National University Health System, Singapore, Singapore; Respiratory Medicine Service, Department of Paediatrics, KK Women’s and Children’s Hospital, Singapore, Singapore; Center for Cognitive Neuroscience, Program in Neuroscience and Behavioral Disorders, Duke-NUS Medical School, Singapore, Singapore; Department of Paediatrics, Yong Loo Lin School of Medicine, National University of Singapore, National University Health System, Singapore, Singapore; Khoo Teck Puat- National University Children’s Medical Institute, National University Health System, Singapore, Singapore; Singapore Institute for Clinical Sciences, Agency for Science, Technology and Research (A*STAR), Singapore, Singapore; Department of Psychiatry, Faculty of Medicine, McGill University, Montreal, Canada; Nestlé Institute of Health Sciences, Nestlé Research, Societé des Produits Nestlé S.A., Switzerland; Office of Education Research, National Institute of Education, Singapore, Singapore; Singapore Institute for Clinical Sciences, Agency for Science, Technology and Research (A*STAR), Singapore, Singapore; Amsterdam UMC and OLVG Location Vrije Universiteit Amsterdam, Department of Psychiatry, Boelelaan 1117, Amsterdam, The Netherlands; Amsterdam Public Health, Mental Health program, Amsterdam, The Netherlands

**Keywords:** sleep trajectory, cognition, sleep duration

## Abstract

**Study Objectives:**

Examine how different trajectories of reported sleep duration associate with early childhood cognition.

**Methods:**

Caregiver-reported sleep duration data (*n* = 330) were collected using the Brief Infant Sleep Questionnaire at 3, 6, 9, 12, 18, and 24 months and Children’s Sleep Habits Questionnaire at 54 months. Multiple group-based day-, night-, and/or total sleep trajectories were derived—each differing in duration and variability. Bayley Scales of Infant and Toddler Development-III (Bayley-III) and the Kaufman Brief Intelligence Test- 2 (KBIT-2) were used to assess cognition at 24 and 54 months, respectively.

**Results:**

Compared to short variable night sleep trajectory, long consistent night sleep trajectory was associated with higher scores on Bayley-III (cognition and language), while moderate/long consistent night sleep trajectories were associated with higher KBIT-2 (verbal and composite) scores. Children with a long consistent total sleep trajectory had higher Bayley-III (cognition and expressive language) and KBIT-2 (verbal and composite) scores compared to children with a short variable total sleep trajectory. Moderate consistent total sleep trajectory was associated with higher Bayley-III language and KBIT-2 verbal scores relative to the short variable total trajectory. Children with a long variable day sleep had lower Bayley-III (cognition and fine motor) and KBIT-2 (verbal and composite) scores compared to children with a short consistent day sleep trajectory.

**Conclusions:**

Longer and more consistent night- and total sleep trajectories, and a short day sleep trajectory in early childhood were associated with better cognition at 2 and 4.5 years.

## Graphical Abstract

**Graphical Abstract:**
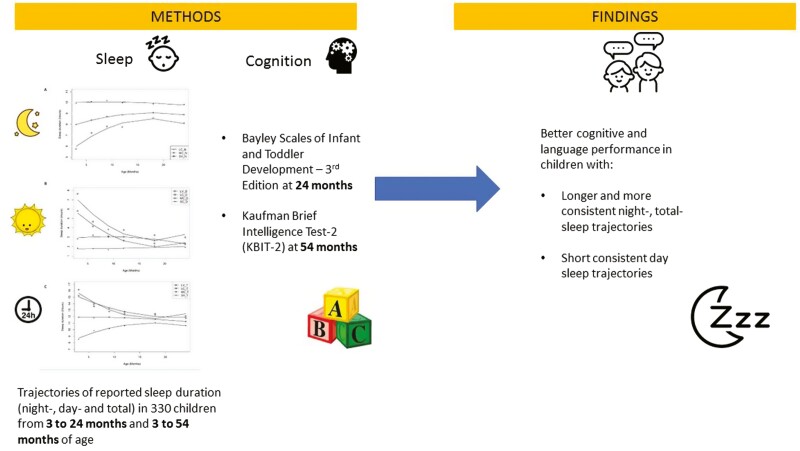

**Statement of Significance**

Sleep duration is associated with better cognitive functioning across the lifespan; however, few studies examined infant sleep and cognitive outcomes and often have cross-sectional designs. To the best of our knowledge, we are the first to derive between individual day-, night- and total sleep duration trajectories and study them in relation to cognitive outcomes in early childhood. In a multi-ethnic Asian population, we show that longer and more consistent night- and total sleep trajectories, as well as a short consistent day sleep trajectory, are associated with better cognitive outcomes. The study adds a longitudinal perspective as well as variability of sleep duration as an important factor to the existing child sleep research. Our findings are important as sleep is potentially modifiable to optimize early development.

## Introduction

Sleep duration is associated with better cognitive functioning across the lifespan and this association starts early in life. Studies have showed that night sleep duration has been associated with better cognitive outcomes in school-aged children [1–4] as well as in adolescents [5, 6] and adults [7, 8]. For example, shorter habitual sleep duration and poor sleep quality have been associated with poorer intelligence quotient (IQ) scores [1] and poorer performance in standardized battery of cognitive tasks [2] in children between 7 and 11 year-olds. Sleep is believed to be important for consolidation of memory [9] and deprivation of sleep has been shown to affect the prefrontal cortex, a brain region involved in executive function and alertness [10]. The proportion of sleep occurring at night has been regarded as a gauge of sleep consolidation in infants and toddlers, with a higher proportion indicating more mature organization of sleep–wake patterns which is linked to neurological maturation and integrity [11, 12]. Studies in infants have associated normative sleep development, sleep consolidation and maturation of sleep–wake patterns to better cognitive outcomes such as better executive function [13–15] and language acquisition [16].

However studies on infant sleep and cognitive outcomes are scarce and unlike the findings from older children, the results revolving around the effects of infant sleep on developmental outcomes are inconsistent [17]. This may be attributed to differences in study designs, with regard to the age at which the sleep was assessed, the timing of developmental test and also the type of infant population sampled (e.g. premature versus term infants) [17]. Another systematic review that focused on sleep and cognition in preschoolers (2–6 years of age) showed that majority of the studies reported modest but significant association between increased sleep quantity and/or quality and better cognitive outcomes [18]. While studies showed that preterm born babies tend to have longer total night sleep duration than term born babies [19] and may be more sensitive to the influence of sleep on cognition [20], results of studies that investigated the relation between sleep duration and cognitive outcomes in term born babies are still inconclusive.

Sleep is a dynamic developmental process, especially during infancy and toddlerhood, with much inter- and intra-variability between children [21–23] over time. However, most studies have used a cross-sectional design [24], with few longitudinal studies on infant sleep and cognition. One prospective study showed that poor infant sleep quality was associated with behavioral problems and poorer attention regulation [25] while another did not see strong link between sleep problems and intelligence quotient (IQ) [26]. It is also of note that a couple of the longitudinal studies were conducted by the same research group, using the same study population of Canadian infants whose sleep were measured at 12 and 18 months and cognition assessed at 18, 26 months [14], and 4 years [13], and both studies reported better executive function with higher proportion of nocturnal sleep. In addition, some of these longitudinal studies explored sleep trajectories using within-individual (single) group trajectories instead of between-individual (multiple) trajectories. While intraindividual variability in sleep has been shown to be associated with various cognitive function in children and adolescents [23], within-individual group trajectory neglects the fact that infants and young children may display different types of developmental sleep trajectories rather than all displaying the same pattern of development. By using multiple trajectories instead of one group trajectory, researchers may be able to identify specific pathways for different types of developmental sleep trajectories in relation to developmental outcomes. For example in a study by Touchette et al. [3], the researchers assessed sleep in children between 2.5 and 6 years old and derived multiple night sleep trajectories (short persistent, short increasing sleep, 10-hour persistent, and 11-hour persistent) and found that only short sleep trajectories, but not the other trajectories, were related to poorer cognitive performance. More recently, Smithson et al. [27] derived night, day, and total sleep duration trajectories of participants from the Canadian Healthy Infant Longitudinal Development (CHILD) birth cohort between 3 and 24 months of age. The authors reported that short sleepers (total sleep) had lower cognitive and language scores as measured by Bayley Scales of Infant Development (Bayley-III) in comparison to intermediate sleepers. They also found that long nocturnal sleepers had higher cognitive and language scores in Bayley-III compared to short and intermediate nocturnal sleepers. Cao et al. observed in a population of Chinese children that children belonging to trajectories characterized by “decreased and then increase nighttime sleepers” and “long and decreased daytime sleepers” between 1 and 24 months of age tend to have lower Bayley-II Mental Development Index (MDI) scores [28]. Apart from cognition, sleep trajectories in early childhood (0–7 years of age) had been associated with health-related quality of life, where persistent short sleepers had reportedly worse emotional, social, and physical health [21].

To the best of our knowledge, between-individual day, night and total sleep trajectories during infancy and early childhood and how they relate to cognitive outcomes is relatively unexplored, although longitudinal studies suggested that greater proportion of night sleep during early childhood is linked to better executive function [13, 14] and language skills [3, 14, 16]. Adding to the study of Touchette et al., which derived longitudinal sleep trajectories based on nocturnal sleep reported by parents between 2.5 and 6 years [3], in this study we derived night, day, and total sleep trajectories based on variability (i.e. standard errors of between-individual differences) and duration of children’s sleep over the ages of 3–54 months [29]. In a multi-ethnic Asian population of term born children, we examine how our derived day, night, and total sleep duration trajectories from early infancy onwards associate with cognitive outcomes over the first 5 years of life. We derived sleep trajectories for 3–24 months as well as 3–54 months to capture the sleep trajectories up till the point of the cognitive tests at 24 and 54 months, respectively. We included day sleep trajectories as infants and toddlers take substantial naps in the day, prior to consolidation of nocturnal sleep [22, 30]. We hypothesized that trajectories with long consistent night and total sleep as well as short consistent day sleep, would associate with better general cognitive performance and development, as measured by Bayley-III at 24 months and the Kaufman Brief Intelligence Test (KBIT-2) at 54 months.

## Methods

### Study population

**Figure 1.**
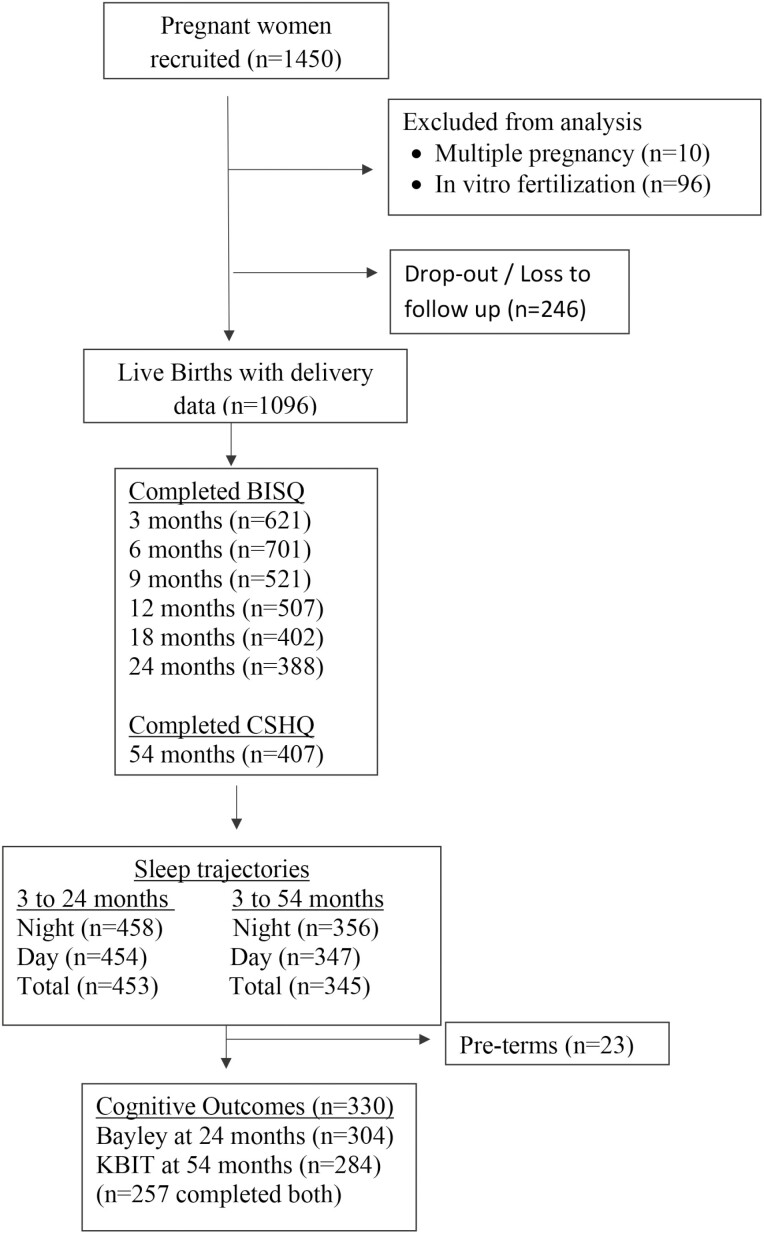
Flow chart describing participant inclusion for the current study.*Had sleep data for at least five timepoints.

Women aged 18 and above were recruited into the Growing Up in Singapore Towards healthy Outcomes (GUSTO) mother–offspring cohort study 31 between June 2009 and September 2010, during their first trimester of pregnancy. Infants in this study were born between November 2009 and May 2011. For this analysis, children who were born preterm (<37 weeks of gestation), conceived by in vitro fertilization, or part of a multiple pregnancy were excluded (Figure 1). We excluded preterm babies for this analysis as they tend to have longer total night sleep duration than term babies [19] and may be more sensitive to the influence of sleep on cognition [20]. We only included participants who had at least one derived sleep trajectory and did at least one cognitive test in this analysis (Figure 1). This study was approved by both the National Health Care Group Domain Specific Review Board (reference D/09/021 and 2014/00414) and the Sing Health Centralized Institutional Review Board (reference 2009/280/D). Informed written consent was obtained from each participant, with procedures approved by the National Healthcare Group Institutional Review Board (IRB) and the SingHealth Centralized IRB.

A total of 330 children had at one or more derived sleep duration trajectories and completed at least one cognitive test (Figure 1). Amongst them, *n* = 257 completed both cognitive tests (Figure 1). The included participants were comparable with those who were excluded, in terms of sex distribution of child and maternal education. The infants included in this analysis had significantly higher gestational age and birth weight, were more likely to be of Indian ethnicity, were breastfed longer and their mothers were significantly older (Table 1). The children included in this analysis had comparable scores for Bayley-III and KBIT-2 with children who were excluded (Table 1).

**Table 1.** Maternal and child characteristics of participants included in this study

	Participants (*n* = 330)	Non-participants (*n* = 766)	*P*
Infant variables			
Gestational age (weeks)	39.1 (1.0)	38.6 (1.7)	<.001
Sex of child (% male)	51.8	52.4	.849
Birth weight (grams)	3147 (394)	3051 (490)	.001
Ethnicity, *n* (%)			
Chinese	191 (57.9)	407 (53.1)	.040
Malay	92 (27.9)	200 (26.1)	
Indian	47 (14.2)	159 (20.8)	
Maternal variables			
Maternal Education, *n* (%)			
Primary/No education	11 (3.3)	34 (4.4)	.201
Secondary	76 (23.0)	221 (28.9)	
Diploma/Technical education	120 (36.4)	264 (34.5)	
University	112 (33.9)	226 (29.5)	
Postgraduate	7 (2.1)	9 (1.2)	
Missing data	4 (1.2)	12 (1.6)	
Maternal age (years)	31.0 (5.2)	30.2 (5.2)	.029
BDI score at 24 months	7.00 ± 7.06	8.34 ± 7.89	.098
BDI score at 54 months	6.07 ± 7.04	7.01 ± 8.22	.239
Breastfeeding duration			
<1 month	64 (19.4)	190 (24.8)	.001
1 to <3 months	57 (17.3)	128 (16.7)	
3 to <6 months	54 (16.4)	105 (13.7)	
6 to <12 months	66 (20.0)	97 (12.7)	
>12 months	84 (25.5)	115 (15.0)	
Missing/unclear	5 (1.5)	131 (17.1)	
Bayley-III scores			
Cognition	10.56 ± 2.61	10.08 ± 2.38	.049
Expressive language	9.18 ± 2.77	9.05 ± 2.73	.614
Receptive language	9.18 ± 2.58	8.99 ± 2.54	.453
Fine motor	10.84 ± 2.32	10.68 ± 2.10	0.477
Gross motor	11.33 ± 3.02	10.85 ± 2.35	0.066
KBIT-2 scores			
Verbal	86.33 ± 16.17	84.76 ± 16.42	.325
Non-verbal	99.99 ± 14.28	98.97 ± 15.84	.484

KBIT, Kaufman Brief Intelligence Test. BDI, Beck Depression Inventory-II.

Data presented as mean (standard deviation) unless otherwise stated.

### Sleep measurements and trajectories

Night, day, and total sleep duration were derived from the caregiver reported Brief Infant Sleep Questionnaire (BISQ) [32] at 3, 6, 9, 12, 18, and 24 months as well as the caregiver reported Children’s Sleep Habits Questionnaire (CSHQ) [33] at 54 months For BISQ, caregivers were asked “How much time (on average) does your child spend in sleep during the night” and “How much time (on average) does your child spend in sleep (naps) during the day”. For CSHQ, caregivers were additionally asked to “Write in child’s bedtime”, “Write in the time-of-day child usually wakes in the morning”, “Write in your child’s usual naptime (if any)” and “Write in the time-of-day child usually wakes after nap (if any)” for both weekends and weekdays. Night and day sleep durations were calculated by taking the average of (5 × weekdays + 2 × weekend)/7. Total sleep durations for both instruments were calculated by summing the day and night sleep durations. Both questionnaires were administered in the dominant language of the caregiver- English, Chinese, Malay, or Tamil language.

**Figure 2.**
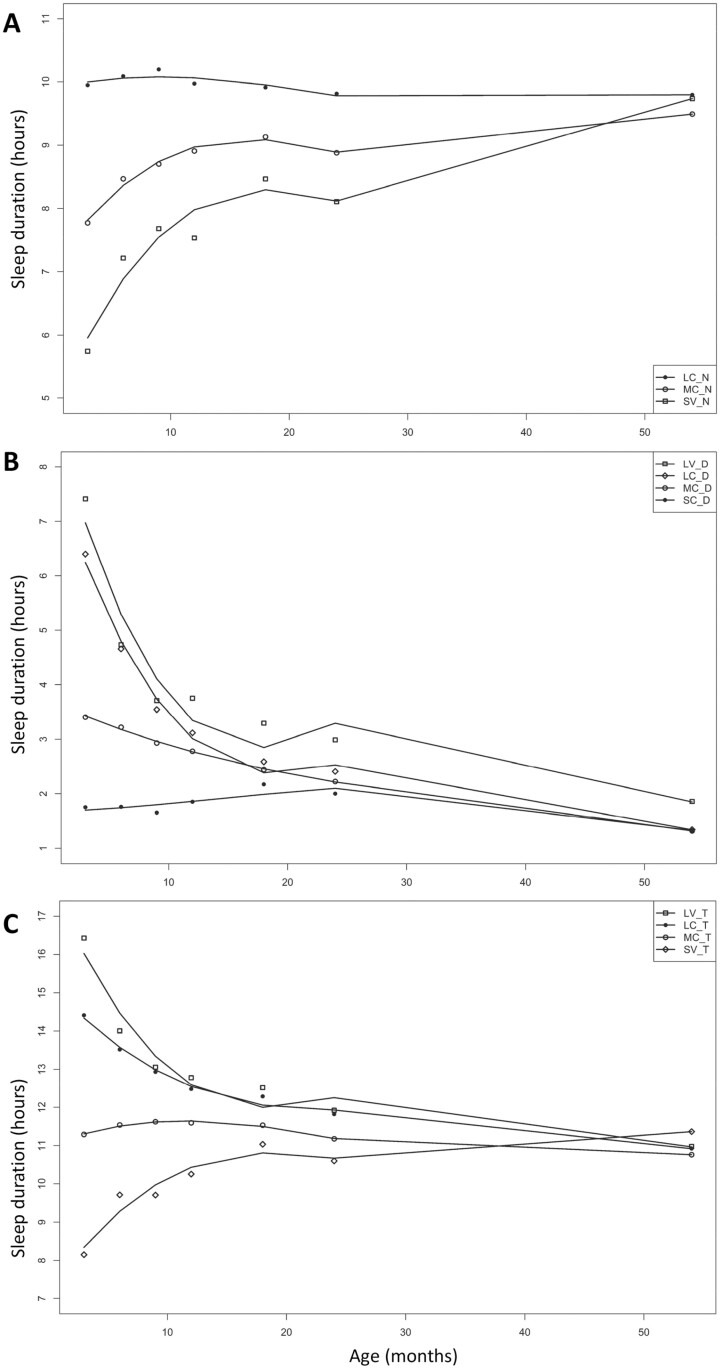
Sleep trajectories from 3 to 54 months. (A) Night sleep duration trajectories. LC_N: long consistent night sleep; MC_N: moderate consistent night sleep; SV_N: short variable night sleep. (B) Day sleep duration trajectories. LV_D: long variable day sleep; LC_D: long decreasing day sleep trajectory; MC_D: moderate consistent day sleep; SC_D: short consistent day sleep. (C) Total sleep duration trajectories. LV_T: long variable total sleep; LC_T: long consistent total sleep; MC_T: moderate consistent total sleep; SV_T: short variable total sleep. Solid lines depict the model trajectory curve. This figure was published in Sleep Health, Vol 7, Issue 1, pages 56–64, Tham et al. (2021), Variations in longitudinal sleep duration trajectories from infancy to early childhood, Page 60–61, Copyright Elsevier (2021).

**Figure 3.**
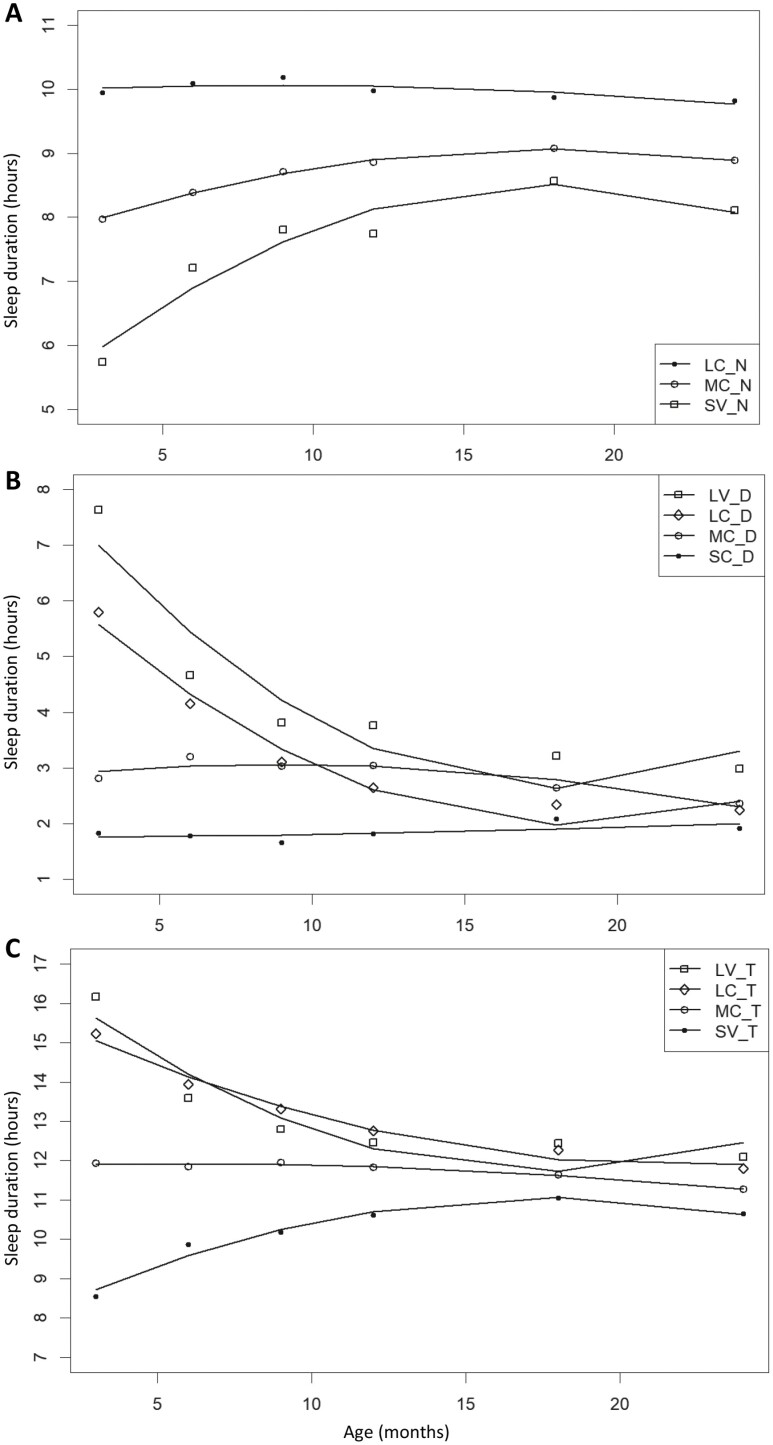
Sleep trajectories from 3 to 24 months. (A) Night sleep duration trajectories. LC_N: long consistent night sleep; MC_N: moderate consistent night sleep; SV_N: short variable night sleep. (B) Day sleep duration trajectories. LV_D: long variable day sleep; LC_D: long decreasing day sleep trajectory; MC_D: moderate consistent day sleep; SC_D: short consistent day sleep. (C) Total sleep duration trajectories. LV_T: long variable total sleep; LC_T: long consistent total sleep; MC_T: moderate consistent total sleep; SV_T: short variable total sleep. Solid lines depict the model trajectory curve.

Multiple sleep duration trajectories were derived for all participants of the GUSTO study with sleep duration data for at least 5 timepoints as per previously described [29]. These sleep trajectories have been based on the caregiver reported BISQ data and CSHQ data. In brief, a multi-step group-based trajectory algorithm was applied to detect night, day, and total sleep trajectories of children between 3 months and 54 months of age. It is a novel conditional probabilistic trajectory model which does not constrain a constant variance among different trajectory groups. Individual based sleep trajectories over time were first extracted through a multiple regression model and the children were grouped using K-means approach without a pre-defined cluster number. Group-based trajectories were initialized considering all individuals in each cluster and children were reallocated based on their likelihood (maximum likelihood value) to group-based trajectories. The optimal number of groups for each sleep trajectory (i.e. day, night, and total sleep) was determined by comparing model Bayesian Information Criterion (BIC) from 5-fold cross validation. Eventually, three night (short variable, moderate consistent, and long consistent), four day (short consistent, moderate consistent, long variable, and long consistent) and four total (short variable, moderate consistent, long variable, and long consistent) sleep trajectories were derived (Figure 2) and differentiated based on duration and variability. Variability refers to the standard errors from the model trajectory curve, with consistent trajectories having smaller standard errors from the model curve, compared to variable trajectories (Figure S1). The above steps were repeated using sleep data from 3 to 24 months to derive new night-, day-, and total sleep trajectories, keeping to the same number of groups as for the 3–54 months trajectories (Figure 3). Although constraining the number of groups did not yield the best-fitting models in terms of BIC, the model fit (measured by average posterior probability of assignment (APPA) [34]) were comparable or better in the models with the fixed number of trajectories. We derived two sets of sleep trajectories (3–24 months and 3–54 months) so as to study the association of sleep patterns up to the point of the cognitive assessments (24 and 54 months). Quadratic curves are applied to generate the 3–24 months sleep trajectories as the observation period is shorter, with minimum four time points of sleep data required to build a quadratic trajectory for each child. All sleep duration trajectories were derived with the R package version 3.4.3 (R Core Team). The number of subjects with sleep data and derived sleep trajectories are outlined in Figure 1.

### Cognitive measurements

A subset of participants agreed to participate in the neurodevelopment assessment at 24 months (*n* = 514) and 54 months (*n* = 491), as described previously [35]. These children completed the Bayley-III at 24 (±1) months of age. Bayley-III is a standardized test with five US age-norm subscales scores. Besides scales for cognition, expressive, and receptive language, Bayley-III also includes fine and gross motor skills and hence considered a “general developmental scale” [35]. At 54 months (+2 months), the children completed the Kaufman Brief Intelligence Test, second edition (KBIT-2) [36], which measures the verbal and nonverbal intelligence to derive a composite IQ score. All tests were administered in the child’s dominant language by research coordinators trained by clinicians from Kandang Kerbau Women’s and Children’s Hospital and all test scores were age standardized. The mean and standard deviation of the Bayley-III and KBIT scores of the study population is included in Table 1. All subscales of Bayley-III and KBIT were significantly correlated (*P* < 0.05) and the Pearson correlation coefficients ranged from 0.132 to 0.555.

### Other data

Demographic data such as maternal education and ethnicity were collected by interviewer administered questionnaires during enrollment. Birth outcomes such as birth weight, sex of the child, and gestational age were recorded by trained midwives and research staff at delivery. During postpartum visits (at 3, 6, 9, 12, 15, 18, 24, 36, and 48 months), participants were asked by interviewers if they were breastfeeding or when they stopped breastfeeding. Beck Depression Inventory-II (BDI-II) were self-administered by the mothers at 24 and 54 months.

### Statistical analysis

Independent *t*-test and chi-square test were used to compare the continuous and categorical child and maternal characteristics, respectively. Multivariable linear regression models were run to assess the associations between the day, night, and total sleep trajectories up to the point of cognitive assessment, on the cognitive performance (i.e. 3–24 months sleep trajectories on Bayley-III and 3–54 months sleep trajectories on KBIT-2). The models were adjusted for covariates that are associated with sleep and are known to affect cognitive outcomes, such as maternal education, birth weight, sex, and ethnicity of the child and breastfeeding duration. The Dunnett post hoc test was used for multiple comparisons with reference to the short trajectory. Short trajectories were used as the reference since several studies before us showed that short sleep trajectories were associated with poorer cognitive outcomes [3, 27]. Sensitivity analyses were done, with additional adjustment for maternal depressive symptoms at the same timepoint as the cognitive testing (i.e. 24 and 54 months, respectively, for Bayley-III and KBIT-2) as maternal mood may influence the reporting of the sleep duration as well as the child’s cognitive outcomes. However, we did not include it as a covariate as it may be on the causal pathway between the sleep trajectories and cognitive outcomes as child sleep patterns have been linked to maternal depressive symptomology [37], which in turn has been linked to child cognitive outcomes [38, 39]. Listwise deletion was used to handle missing data. All analyses were done with SPSS 26.0 (IBM) except the sleep trajectories which were derived using the R package version 3.4.3 (R Core Team) [29].

## Results

### Sleep trajectories

Table 2 shows the distribution of the participants across the various night-, day- and total sleep duration trajectories. Table 3 summarizes the average duration of day-, night-, and total sleep for the various sleep trajectories group (3–54 months) across the time-points. We observed previously that the various day-, night-, and total sleep trajectories start to converge between 24 and 54 months of age [29], suggesting that individual differences in sleep duration mostly occur around infancy and early toddlerhood as they gradually consolidate their sleep. Amongst those who have trajectories for both 3–24 months and 3–54 months, 96.4%, 85.3%, and 81.9% overlapped for night, day, and total sleep, respectively. This suggest that the trends that we observed, in terms of duration and variability, are driven largely by the data from first 24 months. However, significant difference in variability was still observed at 54 months [29].

**Table 2.** Distribution of night-, day-, total sleep trajectories amongst participants analyzed (*n* = 330)

Sleep trajectories	3–24 months	3–54 months
Night		
Long consistent	107 (32.4%)	101 (30.6%)
Moderate consistent	124 (37.6%)	126 (38.2%)
Short variable	99 (30.0%)	82 (24.8%)
Not derived	0 (0.0%)	21 (6.4%)
Day		
Long variable	74 (22.4%)	60 (18.2%)
Long consistent	82 (24.8%)	66 (20.0%)
Moderate consistent	93 (28.2%)	101 (30.6%)
Short consistent	77 (23.2%)	73 (22.1%)
Not derived	4 (1.2%)	30 (9.1%)
Total		
Long consistent	67 (20.3%)	79 (23.9%)
Long variable	59 (17.9%)	59 (17.9%)
Moderate consistent	93 (28.2%)	83 (25.2%)
Short variable	107 (32.4%)	78 (23.6%)
Not derived	4 (1.2%)	31 (9.4%)

Data presented as *n* (%).

**Table 3.** Mean sleep duration for the various day, night, and total sleep trajectories (3 to 54 months)

Sleep trajectories	Age (months)						
	3	6	9	12	18	24	54
***N***	214	232	210	211	203	199	145
Night							
Long consistent	9.8 ± 1.3	10.1 ± 1.2	10.2 ± 1.1	10.0 ± 0.9	9.8 ± 1.0	10.0 ± 0.9	9.8 ± 1.1
Moderate consistent	7.9 ± 1.1	8.5 ± 1.0	8.7 ± 1.0	8.9 ± 0.9	9.1 ± 1.0	8.9 ± 0.8	9.5 ± 0.8
Short variable	5.7 ± 2.0	7.3 ± 2.1	7.7 ± 2.0	7.4 ± 2.1	8.4 ± 1.8	8.2 ± 2.4	10.2 ± 3.4
**Day**							
Long variable	7.2 ± 3.0	5.0 ± 2.7	3.7 ± 1.8	3.7 ± 1.5	3.5 ± 1.7	3.2 ± 1.4	2.0 ± 2.2
Long consistent	6.5 ± 1.2	4.6 ± 0.9	3.3 ± 1.1	3.0 ± 0.9	2.6 ± 0.6	2.4 ± 0.8	1.3 ± 0.9
Moderate consistent	3.3 ± 1.3	3.2 ± 1.1	2.9 ± 1.1	2.8 ± 1.2	2.5 ± 0.8	2.3 ± 0.7	1.2 ± 1.0
Short consistent	1.8 ± 0.8	1.7 ± 0.8	1.6 ± 0.7	1.9 ± 0.7	2.2 ± 0.7	2.0 ± 0.7	1.4 ± 0.8
**Total**							
Long consistent	14.4 ± 1.6	13.4 ± 1.2	13.0 ± 1.2	12.5 ± 1.2	12.4 ± 1.1	12.2 ± 0.9	10.9 ± 0.9
Long variable	15.8 ± 3.3	14.5 ± 2.8	13.1 ± 2.0	12.5 ± 2.6	12.4 ± 2.3	12.1 ± 2.7	10.8 ± 2.4
Moderate consistent	11.2 ± 1.7	11.5 ± 1.3	11.5 ± 1.2	11.6 ± 1.3	11.4 ± 1.1	11.2 ± 1.0	10.7 ± 1.1
Short variable	8.2 ± 2.2	9.7 ± 2.5	9.4 ± 2.2	10.2 ± 2.0	10.8 ± 1.9	10.7 ± 1.5	11.5 ± 2.4

Data presented as mean ± standard deviation.

### Sleep duration trajectories (3–24 months) and Bayley-III at 24 months of age

Children with a long consistent night sleep trajectory had significantly higher cognition and language (expressive and receptive) scores, compared to children with a short variable night sleep trajectory (Table 4). Conversely, children with long variable day sleep trajectory had lower scores on cognition and fine motor subscales relative to those with short consistent day sleep trajectory (Table 5). Long consistent total sleep trajectory was associated with higher cognitive and expressive language scores compared to short variable total sleep trajectory (Table 6). Moderate consistent total sleep trajectory was associated with higher language (expressive and receptive) scores compared to the short variable total sleep trajectory. Findings are similar with adjustment for maternal depressive symptoms at 24 months (data not shown).

**Table 4.** Association between night sleep trajectories (3–24 months) and Bayley Scales of Infant Development scores

	Unadjusted^1^			Adjusted^2^	
	Short variable (*n* = 92)	Moderate consistent (*n* = 109)	Long consistent (*n* = 103)	Moderate consistent	Long consistent
Cognition	10.11 ± 2.67	10.46 ± 2.54	11.08 ± 2.55	0.22 (–0.60 to 1.05)	**1.02 (0.18 to 1.85)**
Expressive language	8.80 ± 2.34	9.11 ± 2.69	9.59 ± 2.62	0.39 (–0.41 to 1.19)	**0.89 (0.09 to 1.70)**
Receptive language	8.62 ± 2.50	9.27 ± 2.87	9.60 ± 2.82	0.74 (–0.11 to 1.59)	**1.09 (0.23 to 1.94)**
Fine motor	10.71 ± 2.20	10.75 ± 2.51	11.04 ± 2.24	–0.01 (–0.78 to 0.76)	0.44 (–0.33 to 1.22)
Gross motor	11.03 ± 3.19	11.06 ± 2.62	11.85 ± 3.20	–0.12 (–1.09 to 0.85)	0.92 (–0.05 to 1.90)

^1^Values are unadjusted means ± SDs.

^2^Adjusted mean difference in Bayley scores (95% confidence interval) from reference group (short variable), adjusted for maternal education, sex, ethnicity, and birth weight of the child and breastfeeding duration.

**Table 5.** Association between day sleep trajectories (3–24 months) and Bayley Scales of Infant Development scores

	Unadjusted^1^				Adjusted^2^		
	Short consistent (*n* = 74)	Moderate consistent (*n* = 84)	Long variable (*n* = 67)	Long consistent (*n* = 75)	Moderate consistent	Long variable	Long consistent
Cognition	10.81 ± 2.55	10.33 ± 2.81	9.87 ± 2.22	11.09 ± 2.60	–0.65 (–1.65 to 0.34)	**–1.11** **(–2.15 to –0.06)**	0.16 (–0.85 to 1.18)
Expressive language	9.03 ± 2.34	9.07 ± 2.60	8.66 ± 2.45	9.84 ± 2.76	–0.16 (–1.12 to 0.81)	0.69 (–1.70 to 0.32)	0.60 (–0.38 to 1.57)
Receptive language	9.26 ± 2.35	9.00 ± 2.73	8.40 ± 2.44	9.88 ± 3.21	–0.56 (–1.58 to 0.45)	–1.06 (–2.13 to 0.01)	0.29 (–0.75 to 1.32)
Fine motor	11.26 ± 2.27	10.71 ± 2.46	10.30 ± 1.72	11.08 ± 2.62	–0.49 (–1.41 to 0.44)	**–1.03** **(–2.00 to –0.06)**	–0.15 (–1.09 to 0.79)
Gross motor	11.51 ± 3.17	11.40 ± 3.01	10.54 ± 2.70	11.79 ± 3.12	0.04 (–1.13 to 1.21)	0.19 (–1.00 to 1.39)	–0.99 (–2.22 to 0.24)

^1^Values are unadjusted means ± SDs.

^2^Adjusted mean difference in Bayley scores (95% confidence interval) from reference group (short consistent), adjusted for maternal education, gender, ethnicity, birth weight, and breastfeeding duration.

**Table 6.** Association between total sleep trajectories (3–24 months) and Bayley Scales of Infant Development scores

	Unadjusted^1^				Adjusted^2^		
	Short variable (*n* = 101)	Moderate consistent (n = 87)	Long variable (*n* = 52)	Long consistent (*n* = 60)	Moderate consistent	Long variable	Long consistent
Cognition	10.06 ± 2.62	10.77 ± 2.62	10.35 ± 2.50	11.17 ± 2.49	0.58 (–0.35 to 1.50)	0.16 (–0.92 to 1.24)	**1.13** **(0.11 to 2.15)**
Expressive language	8.59 ± 2.25	9.54 ± 2.68	9.08 ± 2.70	9.63 ± 2.67	**1.01** **(0.12 to 1.90)**	0.35 (–0.69 to 1.38)	**1.09** **(0.11 to 2.07)**
Receptive language	8.54 ± 2.29	9.75 ± 3.14	8.83 ± 2.57	9.58 ± 2.80	**1.18** **(0.23 to 2.12)**	0.13 (–0.98 to 1.23)	1.02 (–0.02 to 2.06)
Fine motor	10.64 ± 2.37	11.00 ± 2.59	10.71 ± 1.97	11.08 ± 2.16	0.48 (–0.37 to 1.34)	0.04 (–0.95 to 1.04)	0.57 (–0.37 to 1.51)
Gross motor	11.05 ± 3.18	11.42 ± 2.65	11.46 ± 2.91	11.55 ± 3.41	0.52 (–0.57 to 1.61)	0.37 (–0.89 to 1.64)	0.80 (–0.40 to 2.00)

^1^Values are unadjusted means ± SDs.

^2^Adjusted mean difference in Bayley scaled scores (95% confidence interval) from reference group (short variable), adjusted for maternal education, sex, ethnicity, and birth weight of the child and breastfeeding duration.

### Sleep duration trajectories (3–54 months) and KBIT-2 at 54 months of age

Children with either moderate consistent or long consistent night sleep trajectories had significantly higher verbal score and composite IQ score compared to children with short variable night sleep trajectory (Table 7). On the other hand, children with long variable day sleep trajectory had significantly lower verbal and composite IQ scores relative to children with short consistent day sleep trajectory (Table 8). Moderate consistent and long consistent total sleep trajectories were linked to better verbal performance on KBIT in contrast with short variable total sleep trajectory (Table 9). Long consistent total sleep trajectory was also associated with higher composite IQ scores compared to short variable total sleep trajectory. No significant differences were observed with nonverbal KBIT scores between the sleep trajectories relative to the short duration sleep trajectories. Findings are similar with adjustment for maternal depressive symptoms at 54 months (data not shown).

**Table 7.** Association between night sleep trajectories (3 to 54 months) and KBIT-2 scores

	Unadjusted^1^			Adjusted^2^	
	Short variable (*n* = 71)	Moderate consistent (*n* = 113)	Long consistent (*n* = 92)	Moderate consistent	Long consistent
Verbal	81.58 ± 15.51	88.05 ± 15.69	88.27 ± 17.05	**6.94 (1.93 to 11.94)**	**7.28 (2.03 to 12.54)**
Non-verbal	98.13 ± 16.12	100.86 ± 13.62	100.04 ± 13.98	2.08 (–2.68 to 6.84)	1.83 (–3.16 to 6.81)
Composite IQ	88.52 ± 15.50	93.83 ± 14.05	93.63 ± 14.69	**5.20 (0.64 to 9.76)**	**5.42 (0.62 to 10.21)**

^1^Values are unadjusted means ± SDs.

^2^Adjusted mean difference in KBIT scores (95% confidence interval) from reference group (short variable), adjusted for maternal education, sex, ethnicity, and birth weight of the child and breastfeeding duration.

**Table 8.** Association between day sleep trajectories (3 to 54 months) and KBIT-2 scores

	Unadjusted^1^				Adjusted^2^		
	Short consistent (*n* = 64)	Moderate consistent (*n* = 92)	Long variable (*n* = 52)	Long consistent (*n* = 59)	Moderate consistent	Long variable	Long consistent
Verbal	86.33 ± 16.20	88.16 ± 18.15	80.02 ± 11.51	89.71 ± 15.14	1.53 (–4.18 to 7.24)	**–7.16** **(–13.82 to –0.51)**	2.78 (–3.60 to 9.16)
Non-verbal	103.75 ± 13.00	99.34 ± 14.25	97.56 ± 15.21	99.37 ± 13.48	–4.37 (–9.68 to 0.95)	–5.76 (–11.97 to 0.44)	–4.60 (–10.52 to 1.33)
Composite IQ	95.53 ± 14.46	93.15 ± 15.55	87.19 ± 11.62	93.93 ± 14.04	–1.53 (–6.67 to 3.62)	**–7.59** **(–13.58 to –1.59)**	–1.08 (–6.82 to 4.67)

^1^Values are unadjusted means ± SDs.

^2^Adjusted mean difference in KBIT scores (95% confidence interval) from reference group (short consistent), adjusted for maternal education, sex, ethnicity, and birth weight of the child and breastfeeding duration.

**Table 9.** Association between total sleep trajectories (3–54 months) and KBIT-2 scores.

	Unadjusted^1^				Adjusted^2^		
	Short variable (*n* = 71)	Moderate consistent (*n* = 74)	Long variable (*n* = 49)	Long consistent (*n* = 72)	Moderate consistent	Long variable	Long consistent
Verbal	80.52 ± 15.21	88.89 ± 15.02	83.24 ± 13.77	92.33 ± 17.58	**8.22** **(2.41 to 14.03)**	2.12 (–4.49 to 8.74)	**11.50** **(5.60 to 17.40)**
Non-Verbal	99.86 ± 15.61	100.47 ± 11.51	100.67 ± 16.00	99.33 ± 13.84	0.77 (–4.71 to 6.25)	1.64 (–4.60 to 7.88)	–0.63 (–6.15 to 4.89)
Composite IQ	88.94 ± 15.64	94.09 ± 12.56	90.84 ± 2.00	95.56 ± 14.77	5.16 (–0.15 to 10.46)	2.09 (–3.96 to 8.13)	**6.38** **(1.00 to 11.76)**

^1^Values are unadjusted means ± SDs.

^2^Adjusted mean difference in KBIT scores (95% confidence interval) from reference group (short variable), adjusted for maternal education, sex, ethnicity, and birth weight of the child and breastfeeding duration.

## Discussion

To the best of our knowledge, this study is among the first to investigate associations between-individual (multiple) day, night and total sleep duration trajectories over early childhood (3–54 months) and investigate their associations with cognitive development up to 54 months. We observed that night and total sleep trajectories that are relatively consistent and moderate or long in duration are associated with better cognitive and language outcomes compared to short variable night and total sleep trajectories. Short consistent day sleep trajectory is associated with better cognitive, verbal, and fine motor development compared to longer and more variable day sleep trajectories, especially long variable day sleep trajectory. These trends are observed with 3–24 months as well as 3–54 months sleep trajectories. Coupled with the large overlap between the two sets of sleep trajectories (3–24 months and 3–54 months), we can infer that the sleep trajectories and their associations with neurodevelopmental outcomes are largely driven by differences in sleep patterns in the first 24 months.

Only a couple of studies used longitudinal trajectories of sleep duration during early childhood and examined them in relation to cognitive outcomes [3, 27]. Touchette et al. [3] found short night sleep trajectories, between the age of 2.5 and 6 years, to be associated with higher likelihood of poor language acquisition (as measured by the Peabody Picture Vocabulary Test revised—PPVT-R) and general cognitive performance (Block Design of the Wechsler Intelligence Scale for Children- WISC-III). While the poorer language performance with PPVT (at 5 years of age) is similar to our findings with the KBIT-2 verbal scale of approximately the same age, we observed significant difference in cognitive performance only at 24 months (Bayley-III cognitive scale) but not 54 months (KBIT-2 non-verbal scale). The difference in findings could be attributed to the difference in nocturnal sleep trajectories between their study and ours. The lack of sleep data between 24 and 54 months may have prevented us from deriving two different short sleep trajectories as reported by Touchette et al. It is also noteworthy that 10 and 11 hour persistent sleepers sleep approximately 1 hour more than our moderate- and long consistent night sleepers.

Similar to our study, Smithson et al. identified 4 total-, 3 night-, and 4 day-sleep duration trajectories from the Canadian Healthy Infant Longitudinal Development (CHILD) birth cohort during early childhood (from 3 months to 2 years of age). At 2 years of age, they found that for total sleep trajectory, short sleepers had lower scores on the Bayley-III cognitive and languages scales compared to intermediate sleepers. Similarly, children with short and intermediate night sleep trajectories fared poorer on both cognitive and language scales compared to children with long night sleep trajectories. Their findings are consistent with our findings, in that children from long and consistent total and night sleep trajectories performed better in both Bayley-III cognitive and language scales compared to children from short total and night sleep trajectories. Separately, Cao et al. [28] derived 4 total-, 4 night-, and 3 day-sleep trajectories using 1–24 months sleep data from a birth cohort in China and found that a “decreased then increased” night trajectory was linked to poorer performance on the Bayley-II MDI. However, they did not observe significant differences in Bayley-II performance between the total sleep trajectory groups. It is important to note that the mean total sleep duration of our short sleep trajectory (up to 24 months) and intermediate sleep trajectory (up to 9 months) did not meet the National Sleep Foundation (NSF) recommendations [40] while both long trajectories were in the middle range of sleep duration recommendations. On the other hand, all the total sleep trajectories derived by Smithson et al. [27] from the CHILD cohort, had longer sleep duration than ours, with only the very early portion of the short trajectory (up to 9 months) and intermediate trajectory (3 months) not meeting the NSF recommendations. Their long trajectory is on the high end of sleep duration recommendations. The intermediate total sleep trajectory derived by Smithson et al is relatively most similar to our long consistent total sleep trajectory, with both being the only total sleep trajectories in each study which are predominantly within the NSF recommendations and with less variability over time (i.e. more consistent). This may explain the similarity in findings observed in both studies between total sleep trajectory and cognition and language measured by Bayley-III. On the other hand, Cao et al. total sleep trajectories are longer in duration compared to ours and Smithson et al., largely within (or exceed) NSF recommendation. This might explain their lack of significant findings on the Bayley-II between the total trajectory groups.

Our finding of lower cognitive and fine motor scores on Bayley-III with the long variable day sleep trajectory (relative to the short consistent day sleep trajectory) was similar to the lower MDI scores observed by Cao et al. with the “long and decreased” day sleep trajectory [28]. Longer day sleep might lead to later bedtime, poorer night sleep quality and shorter nocturnal sleep [41], hence impairing neurodevelopment. In the CHILD study, Smithson et al. did not see significant differences in cognitive and language development at 2 years between day sleep trajectories [27].

The Bayley-III scaled and KBIT-2 standardized scores were mostly comparable to the US norms (i.e. mean of 10 and 100, respectively) except for language and verbal scales which are slightly lower. This could be attributed to the large proportion of bilingual children in our cohort [42] which is typical of the Singaporean population and has been shown to affect language development compared to monolingual children [43].

Although few studies investigated night, day or total sleep trajectories during early childhood, several groups studied the proportion of total sleep happening at night during early childhood and have linked greater proportion of night sleep to better executive function [13, 14] and language skills [3, 14, 16]. Consistent with most of the literature, we also observed that young children with greater amount of night sleep throughout infancy and early childhood (i.e. long consistent night sleep trajectory), had higher language scores as well as better cognitive performance skills. However, day sleep in early childhood is less investigated in relation to cognitive performance. Plancoulaine et al. found that day/night sleep ratio was positively associated with Full Scale IQ scores [44] Knowland et al. also showed that more night sleep relative to day sleep, was predictive of better receptive vocabulary [45]. Napping has been associated with shorter night sleep and poorer sleep quality [41] and increased napping has been previously reported to negatively affect language comprehension [46]. In our study, day and night sleep durations are inversely correlated, although not significantly. Similar to the findings in Generation R on napping [46], we found a link between long day sleep trajectory and poorer language performance. We found that long variable day sleep trajectory was associated with lower verbal and composite KBIT-2 scores compared to short consistent day sleep trajectory. It is possible that the children who have short consistent day sleep trajectory have early consolidation of night sleep and maturation of sleep–wake rhythms, which have been linked to neurological maturity [11, 12] and associates with better cognitive outcomes [3, 14, 47, 48].

Interestingly, we observed that apart from duration, variability in the sleep trajectories also altered the association with cognitive outcomes. For example, long variable- but not long consistent day sleep trajectory, had significantly lower verbal and composite KBIT-2 scores than short consistent day sleep trajectory. Similarly, we observed that long consistent- but not long variable total sleep trajectory, is associated with higher cognition and language scaled scores on the Bayley-III, as well as higher verbal and composite KBIT-2 scores compared to the short variable total sleep trajectory. Overall, consistent sleep trajectories tend to be associated with better cognitive outcomes compared to variable ones, possibly because they are more typical of the sleep patterns of the sample population (model trajectory curve), without big deviations at one or more timepoints. This is in agreement with a systematic review which suggests that intra-individual variability in children and adolescents is associated with several aspects of cognitive function [23].

Our findings are consistent with systems consolidation models [49] of declarative learning, which hypotheses that during wakefulness, declarative knowledge is (weakly) encoded by the hippocampus and that during sleep these memories are strengthened and transferred into the long-term neocortical networks. Therefore, children with longer habitual sleep durations throughout development have greater opportunities for the transfer of short-term hippocampal memories into long-term neocortical knowledge. It will be interesting if future work using sleep polysomnography can explore if specific sleep phases and components of sleep play an active role in benefiting verbal IQ in preschoolers, as existing studies have shown that slow-wave sleep and sleep spindle activity are important toward learning new words [50, 51]. The main strength of our study is the use of longitudinal sleep data from 3 to 54 months to derive sleep duration trajectories, which better reflect the dynamic development of sleep during infancy and early childhood. Another strength of our study is the inclusion of breastfeeding duration in our model, which is an important confounder associated with both cognition and sleep. We also add on to Cao et al. [28] and Smithson et al. [27] studies by following up with another cognitive test at 54 months, to examine if the cognitive and verbal/language advantage persists. We recognize that our sample size is relatively small and may require replication. We also acknowledge that the participants in our cohort may not be generalizable to the Singapore population or other populations as they were recruited from two hospitals in Singapore, although they are the two largest maternity hospitals and include both private and subsidized patients. Another limitation is that our sleep trajectories were based on subjective caregiver reports of their children’s sleep, which may differ from objective sleep data. Of note, we switched from BISQ (3–24 months) to the age appropriate CSHQ at age 54 months and while parents reported actual sleep duration for BISQ, the sleep duration reported for CSHQ did not take into consideration wake after sleep onset and may introduce some inconsistency in the way the sleep duration was reported at 54 months. We acknowledge that the long gap between the last two timepoints (24 and 54 months) is a limitation and may affect the accuracy of the classification of the 3–54 months sleep trajectories, especially with regard to the variability. While we adjusted for a large number of covariates, we cannot rule out residual confounding by other important factors such as care arrangement or family structure.

In conclusion, our study suggests that the between-individual variations in the sleep duration trajectories across early childhood, starting as early as 3 months of age, play a role towards subsequent cognitive development. The study adds a longitudinal perspective as well as variability of sleep duration as an important factor to the existing child sleep research. Future research is required to determine if there is a causality link between longitudinal sleep duration patterns and cognitive development in early childhood.

## Supplementary Material

zsac264_suppl_Supplementary_Figure_S1

## Data Availability

The data underlying this article will be available upon reasonable request to the corresponding author.
