# Supplementary material for: Trajectories of reported sleep duration associate with early childhood cognitive development
Source: Sleep. 2022 Nov 10;46(2):zsac264. doi: 10.1093/sleep/zsac264 (PMC9905782; doi:10.1093/sleep/zsac264)
Supplement: zsac264_suppl_Supplementary_Figure_S1 [file zsac264_suppl_supplementary_figure_s1.docx]

**Title:** Trajectories of reported sleep duration associate with early childhood cognitive development

**Abbreviated title:** Childhood sleep trajectories and cognition

**Authors and Affiliations:** Shirong Cai^1 2^; Elaine Kwang Hsia Tham^1^; Hai-Yan Xu^3^; Xiuju Fu^3^; Rick Siow Mong Goh^3^; Peter D Gluckman^1 4^; Yap-Seng Chong^1 5^; Fabian Yap^6^; Lynette Pei-chi Shek ^1^ ^7 8^; Oon Hoe Teoh^9^; Joshua J Gooley^9^; Daniel Yam-Thiam Goh^7 8^; Michael J Meaney^1^ ^11^; Nora Schneider ^12^; Anne Rifkin-Graboi^13^; Birit FP Broekman ^1,14,15^

**^1^Singapore Institute for Clinical Sciences,** Agency for Science, Technology and Research (A*STAR) ^2^Human Potential Translational Research Programme, Yong Loo Lin School of Medicine, National University of Singapore ^3^Institute of High Performance Computing, Agency for Science, Technology and Research (A*STAR) ^4^Liggins Institute, University of Auckland ^5^Department of Obstetrics and Gynaecology, Yong Loo Lin School of Medicine, National University of Singapore ^6^Department of Paediatric Endocrinology, KK Women's and Children's Hospital ^7^Department of Paediatrics, Yong Loo Lin School of Medicine, National University of Singapore, National University Health System ^8^Khoo Teck Puat- National University Children’s Medical Institute, National University Health System ^9^Respiratory Medicine Service, Department of Paediatrics, KK Women’s and Children’s Hospital ^10^Center for Cognitive Neuroscience,Program in Neuroscience and Behavioral Disorders,Duke-NUS Medical School ^11^Department of Psychiatry, Faculty of Medicine, McGill University, Montreal, Canada ^12^ Nestlé Institute of Health Sciences, Nestlé Research, Societé des Produits Nestlé S.A. , Switzerland ^13^Office of Education Research, National Institute of Education, Singapore ^14^ Amsterdam UMC and OLVG location Vrije Universiteit Amsterdam, Department of Psychiatry, Boelelaan 1117, Amsterdam, The Netherlands . ^15^ Amsterdam Public Health, Mental Health program, Amsterdam, The Netherlands

**Corresponding author:**

Birit FP Broekman (email: b.broekman@amsterdamumc.nl)

Amsterdam UMC -Location Vrije Universiteit Amsterdam, Department of Psychiatry
Boelelaan 1117, 1081 HV, Amsterdam, Netherlands

Supplementary Figure 1


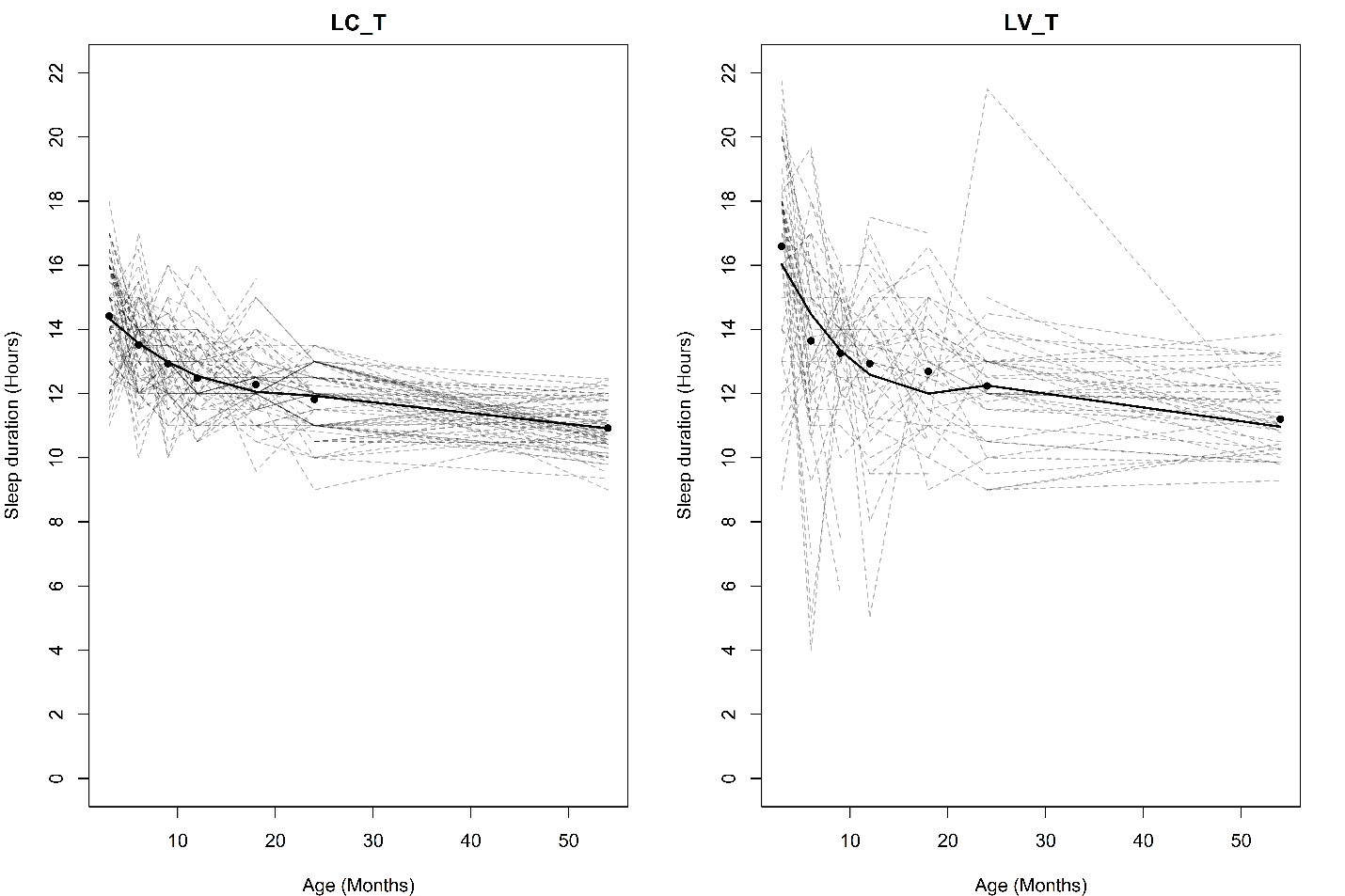


Fig S1. Example of consistent versus variable trajectories. LC_T: long consistent total sleep and LV_T: long variable total sleep. Solid lines depict the model trajectory curve and solid circles depict the study time points. Dotted lines depict individual trajectories.

This figure was published in Sleep Health, Vol 7, Issue 1, Tham et al (2021), Variations in longitudinal sleep duration trajectories from infancy to early childhood, Page 59, Copyright Elsevier (2021).
